# Influence of recording instrumentation on measurements of voice in sentence contexts: use of smartphones and tablets

**DOI:** 10.3389/fdgth.2025.1610772

**Published:** 2025-07-09

**Authors:** Shaheen N. Awan, Yael Bensoussan, Stephanie Watts, Micah Boyer, Robert Budinsky, Ruth H. Bahr

**Affiliations:** ^1^ School of Communication Sciences and Disorders & The Communication Technologies Research Center, University of Central Florida, Orlando, FL, United States; ^2^ Department of Otolaryngology—Head Neck Surgery, University of South Florida Morsani College of Medicine, Tampa, FL, United States; ^3^ Department of Communication Sciences and Disorders, University of South Florida, Tampa, FL, United States

**Keywords:** voice analysis, acoustic analysis, mobile devices, cepstral analysis, spectral tilt, background noise, frequency response

## Abstract

**Introduction:**

The Bridge2AI-Voice. Consortium is developing affordable and accessible voice data to assist in the identification of vocal biomarkers of disease in adults and children. Initial experiments were designed to establish voice recording procedures to be used in research labs and clinical settings, as well as in quiet environments outside of the clinic. The focus has been on isolated vowel productions, which provide a vocal signal that is representative of the biomechanics of the larynx within a static vocal tract. The current experiment considers the impact of sentence productions on the measurement of several acoustic parameters.

**Methods:**

Voice recordings from 24 individuals representing a wide range of typical and disordered voices were analyzed. Two CAPE-V sentences were recorded via a head-and-torso model using (1) a research quality, clinical standard microphone/preamplifier/audio interface and (2) smartphones and tablets using their internal microphones and an attached external headset microphone. Mouth-to-microphone distances and environmental noise levels were controlled. Measures of fundamental frequency (F_0_) and spectral and cepstral measures of voice quality valid for use in sentence contexts were analyzed across recording conditions.

**Results:**

Cepstral peak prominence (CPP) values were sensitive to microphone type, noise, and sentence type conditions. Nevertheless, strong linear relationships were observed across recording methods compared to the clinical standard. Measures of F_0_ obtained using autocorrelation correlated strongly across recording methods, whereas F_0_ measures obtained from the CPP (CPP F_0_) were highly variable and poorly correlated across recording methods and noise conditions. The L/H ratio (a measure of spectral tilt) was significantly affected by recording condition but not background noise, and measures of L/H ratio were also observed to correlate strongly across recording methods and noise conditions.

**Discussion:**

Current findings revealed that different recording methods can produce significantly different acoustic measures of voice with sentence-level materials. Since microphone characteristics (e.g., frequency response; use of noise cancellation), mouth-to-microphone distances, and background noise conditions can have significant effects on spectral and cepstral assessment of voice, it is essential that recording methods and conditions are explicitly described when designing voice data collection projects and comparing datasets as it may have an impact on voice analysis. Future investigations should evaluate consistency of results among multiple examples of the same device.

## 1 Introduction

To evaluate voice quality, clinicians assess voice production in a variety of voice conditions: sustained vowels, reading of specific sentences and/or use of a standardized passage, and conversational speech. Each type of voice sample is believed to represent a different aspect of voice production (1–3). Isolated vowels are easy to produce and analyze because they are free from the influences of phonetic context, intonation, and stress. On the other hand, conversation and reading passages provide insight into how voice quality is impacted by the presence of voiced vs. voiceless sounds, speech timing, and transitions among phonemes. These aspects can alter the perception of voice quality but are believed to be more representative of voice production in a more natural context (2, 4, 5). In addition, certain voice disorders, such as adductor laryngeal dystonia (ADLD; a.k.a. adductor spasmodic dysphonia), may have more severe voice quality disruption during connected speech vs. sustained vowel productions. These disruptions are likely due to the increased linguistic complexity and rapid articulatory adjustments demanded by the speaking task. Therefore, it is recommended that clinicians use both types of voice samples in the assessment of voice disorders.

In addition to a perceptual evaluation of voice, clinicians rely on acoustic analysis to quantify the severity of voice quality disorder by measuring aspects related to pitch, loudness, and quality (2, 3). Typical measurements include fundamental frequency (F_0_), which is associated with the pitch of one's voice. The amplitude of the vocal signal influences the perceived loudness of the voice and measurement of spectral characteristics (i.e., the distribution of energy over time) and signal perturbation provide measures related to voice quality. These acoustic measurements are valuable in classifying vocal disorders and serving as treatment outcome measures following surgical intervention and/or voice treatment.

Research has shown that the utility of acoustic measures in the determination of the severity of vocal pathology will vary by the nature of the voice sample. Parsa and Jamieson (3) considered the use of jitter, shimmer, long-term average spectrum (LTAS), harmonics to noise ratio (HNR), and linear predictive models (including pitch amplitude and spectral flatness ratings) in their measurement of voice quality. They determined that perturbation measures were more reliable when measured with isolated vowels than with connected speech and that linear predictive models were better indicators of voice quality for both isolated vowels and running speech. Their conclusion was that acoustic measurement using continuous speech is most reliable when the voiced sections of speech are separated from the unvoiced sections and pauses.

In another study, Moon et al. (2) found that the acoustic analysis of isolated vowels and sentences resulted in different findings within individuals and by gender. They concluded that the values obtained in connected speech were better representations of the individual's speaking voice as opposed to isolated vowels. However, Gerratt et al. (1) found that the acoustic values for the central portion of isolated vowels and continuous speech were essentially the same. They were careful to explain that one must control the variability in continuous speech associated with different speech contexts and prosodic variations. Their results suggested that isolated vowels generate a less complicated signal to analyze, while the acoustic variations noted in continuous speech can provide more insight into unique aspects of speech production that may be associated with different vocal pathologies.

Measurement of voice quality can also be affected by the type of microphone used for data collection. Microphones capture the voice signal and change it into an electrical signal that can be processed digitally. The response frequency can influence the precision of the acoustic measurement. Titze and Winholtz (6) found that the distance and angle of the microphone in relation to the mouth introduced variability into the recordings used for perturbation measures. In addition, a cardioid microphone, where acoustic information is gathered around the front of the microphone, is preferred to an omnidirectional microphone. Parsa et al. (7) demonstrated that the frequency response of a microphone affected acoustic measurement of vocal parameters, concluding that these recording variations may affect the classification of typical vs. disordered voices.

More recent work by the Bridge2AI Voice Consortium (8, 9) has demonstrated that low-cost headsets, smartphones, and tablets can be used for recording typical and dysphonic voice samples when the microphone to mouth distance is controlled, even in noisy clinic-like environments. Though certain measurements can be significantly affected by recording method and background noise, measurements of F_0_, F_0_ sd, jitter, shimmer, HNR, cepstral peak prominence (CPP), and spectral tilt taken from isolated vowel samples were found to correlate highly with the same measurements made with a lab quality microphone (9). The current investigation expands our previous analyses to include acoustic data obtained from sentence-level materials. The voice samples used in this project were recorded on smartphones and tablets with and without a headset microphone in four different noise conditions. Results are compared to acoustic data obtained from a lab quality microphone. The goal is to establish the utility of using sentence level materials in the assessment of voice quality obtained from smartphones and tablets.

## 2 Method

This study was approved by the University of South Florida Institutional Review Board: Study #004363 *Developing Standards of Acoustic Data for Voice as a Biomarker of Health*. This experiment focuses on the sentence-level data obtained during the data collection for recordings made on smartphones and tablets [see Awan et al. (8, 9) for a more detailed explanation of the data collection procedures].

### 2.1 Voice samples

Audio recordings from 24 individuals representing a range of vocal severity were selected: typical voice quality (*n* = 6), and mild (*n* = 6), moderate (*n* = 6), and severe (*n* = 6) examples of dysphonia. The 16 adult samples (8 females and 8 males) were obtained from the Perceptual Voice Qualities Database (10) and an additional 8 child voices (4 females and 4 males) recorded by the first author (S.A.). The mean age of the individual voices was 39.83 years (age range = 6–88 years) with a mean CAPE-V severity rating of 44.98 (SD = 32.25; range = 2.5–98.67).

### 2.2 Sentence-level stimuli

Recordings of two sentences taken from the Consensus Auditory-Perceptual Evaluation of Voice [CAPE-V (11)] were obtained from individuals representing a wide range of typical and disordered voices. The first sentence, *The blue spot is on the key again* is used to examine the coarticulatory influence of three vowels (/a, i, u/) (11) and contains several voiced and unvoiced stop plosive and fricative productions. The second sentence, *We were away a year ago*, features all voiced sounds and assesses one's ability to maintain voicing across word productions. These voice samples served as the vocal productions to be re-recorded with various smartphones and tablets both directly using their built-in microphones and with external headset microphones at comfortable distances per device and in a variety of noise levels.

### 2.3 Recording test procedures

A head-and-torso (HAT) model (GRAS 45BC-12 KEMAR; GRAS Sound Vibration USA, Beaverton, OR) was used to present the sentence stimuli at an intensity equivalent to 67 dB C at 30 cm (which is consistent with a normative expectation of 65–70 dB C for a typical speaking voice). Recording levels were set to peak at approximately −12 dB FS (full scale). Voice samples were recorded using a research quality standard microphone/preamplifier/audio interface (GRAS 40AF Free-Field Microphone + GRAS 26AK ½′′ Microphone Preamplifier + GRAS 12AA 2-Channel Power Module [GRAS Sound Vibration USA, Beaverton, OR] + Focusrite Scarlett 2i2 3rd Gen USB Audio Interface [Focusrite Audio Engineering Ltd., High Wycombe, Bucks, United Kingdom]), as well as two smartphones (Apple iPhone 13 Pro [Model MLTQ3LL/A, iPhone OS v. 15.2] and Google Pixel 6 [Model GB7N6, Android version 12]) and two tablets (Apple iPad [9th Generation; Model A2602; iPad OS v. 17.3.1] and a Samsung Galaxy Tab A8 [Model SM-X200; Android v. 13.0]). Smartphone and tablet recordings were captured in two ways: (a) direct using the built-in tablet microphones [capacitive microelectromechanical systems (MEMs) condenser microphones] and (b) using an Avid AE-36 (AVID Products, Inc., Middletown, RI) low-cost headset microphone. The Avid AE-36 uses an electret condenser microphone that requires approximately 5v phantom power. It is omni-directional and noise-canceling and validated in previous work (8, 9, 12). The justification for choosing the AVID headset microphone dealt primarily with the ability to provide a headset microphone to multiple recording sites for large-scale voice data collection to be used in voice AI research. Therefore, cost, size, availability, and ease of use with children were key considerations.

**Figure 1:**
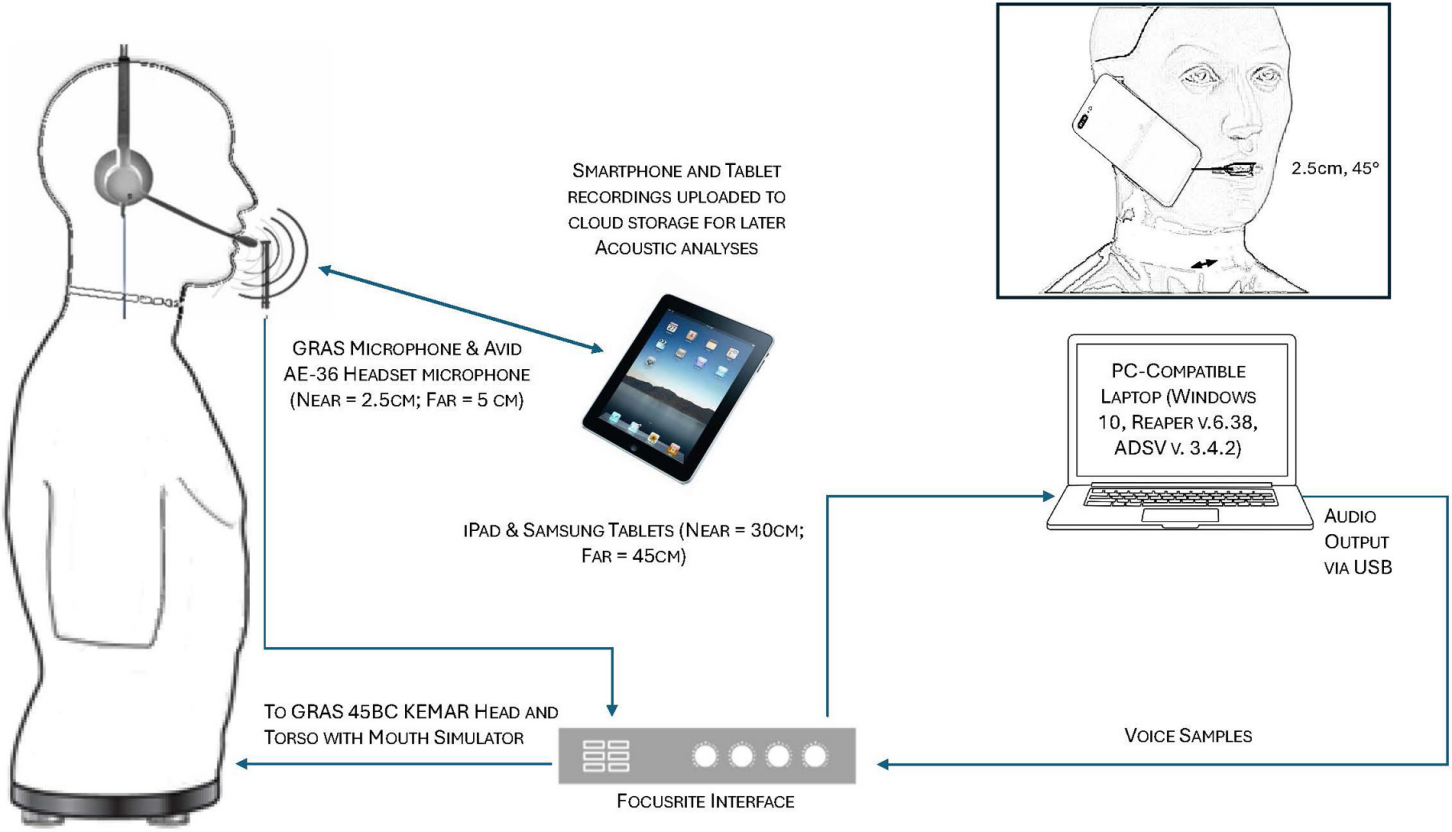
Schematic of the voice sample playback and recording setup for **(a)** the GRAS standard microphone, **(b)** headset microphone recordings, and **(c)** tablet recordings. In addition, the inset figure (top right) shows the smartphone positioning in relation to the mouth opening of the head and thorax (HAT) model. For the purposes of illustration clarity, distances are not to scale.

Recording distances were selected based on what were considered as comfortable and typical use distances. For smartphone direct recordings using the internal smartphone microphone, a smartphone holder (Hercules DG307B, Hercules Stands) was attached to a boom microphone stand with the smartphones positioned in a natural position against the HAT ear and at 2.5 cm (≈1 in.) from the HAT mouth opening at an approximate 45° offset. Tablet-direct recordings were conducted at comfortable arm lengths at 30 and 45 cm at an angle of approximately 30 degrees from the HAT mouth opening. For recordings using smartphones and tablets with the Avid AE-36 headset microphone, the microphone was placed at 2.5 cm from the HAT mouth opening at an approximate 45° offset. Microphone recordings were captured at 44.1 kHz, 16 bits using *Reaper* v.6.78 (13) on a Windows 10 laptop. All tablet recordings were similarly captured at 44.1 kHz, 16 bits using the Shure Motiv audio recording app (Shure Incorporated, Niles, IL). Separate recording sessions were conducted for each tested device so that HAT mouth-to-microphone distances and angles could be standardized. Figure 1 provides an illustration of the recording setup for the various devices and detailed descriptions of the methodology used for smartphone and tablet recordings with and without the use of the headset microphone are also provided in Awan et al. (8, 9).

### 2.4 Frequency response characteristics

The frequency response characteristics of the Avid AE-36 headset microphone and the built-in smartphone (iPhone 13 and Google Pixel 6) and tablet (iPad and Samsung Galaxy Tab) microphones were previously reported in Awan et al. (8, 9) and assessed by subtracting the response of a flat-response reference microphone to a pink noise signal from each device under test (14). The Avid microphone has been reported to be relatively flat (±3 dB) from 50 Hz to approximately 5,000 Hz, followed by a high-frequency emphasis in the region of 8,000–10,000 Hz. Both smartphones (iPhone 13 and Google Pixel 6) showed similar response curves with +2–3 dB emphasis observed at approximately 2.5 kHz, followed by a high-frequency emphasis > 7 kHz. The Samsung tablet microphone direct was observed to have a very similar profile to the Avid microphone, while the iPad was observed to be relatively flat from approximately 75–10,000 Hz. No proximity effect was observed for the smartphones, tablets, or the AVID AE-36 headset microphone. The mean RMS dB FS noise levels of the headsets and built-in smartphone and tablet microphones were approximated from segments of audio silence during sound-attenuating booth recordings. All noise levels were observed to be substantially less than 10 dB below the sound level of the quietest phonation in the voice sample corpus as recommended by Patel et al. (15).

### 2.5 Noise conditions

Recordings made in the sound-attenuating booth had an ambient level of 44 dB C. To simulate clinical conditions, recordings were made with added background noise obtained from three typical examination rooms in the University of South Florida Voice Health Center: a voice clinic office [Exam Room 1 (ER1); average ambient noise level = 54.6 dB C]; a stroboscopy clinic room housing an Olympus (Olympus America, Center Valley, PA) stroboscopy system [Exam Room 2 (ER2); average noise level = 58.9 dB C]; and a stroboscopy clinic room housing a Pentax (Pentax Medical Inc., Montvale, NJ) stroboscopy system [Exam Room 3 (ER3); average ambient noise level = 58.0 dB C]. These recordings were collected with LED lighting and equipment turned on (as would be present during a complete clinical voice evaluation). Long-term average spectrum (LTAS) analyses of the background noise recordings were conducted with ER1 showing an increase in spectral energy due to background noise of ≈10 dB in the 0–1,000 Hz region and an increase of ≈5–7 dB above 4,000 Hz vs. booth recording. ER2 and ER3 showed an increase in spectral energy due to background noise of ≈15 dB in the 0–1,000 Hz region, an increase of ≈5–7 dB above 4,000 Hz, and peaks of ≈ + 20 dB vs. the booth recording at 1,000 Hz (ER2) and 2000 Hz (ER3) (8, 9). During voice sample recording, these background noise samples were played simultaneously with KEMAR voice sample playback via speakers (KEF Q100 Model 3722) positioned at 0°, 90°, 180°, and 270° at a distance of 1 m.

### 2.6 Acoustic and statistical analyses

Sentence samples obtained via the various recording methods were analyzed using *Praat* (16) scripts by Heller Murray (17) and Awan et al. (8, 9) for the following measures of vocal frequency and quality: mean fundamental frequency (F_0_ Hz) computed using two methods (Method 1: CPP F_0_—F_0_ estimated from the quefrency location of the detected cepstral peak); Method 2: F_0_ estimated using the “Analyze Periodicity | To Pitch (raw autocorrelation…” method in *Praat* recommended for intonation analyses); cepstral peak prominence (CPP dB; the amplitude of the cepstral peak in relation to a linear regression line computed though the cepstrum, computed without voice activity detection); and the low vs. high spectral ratio (L/H ratio in dB using a 4 kHz cutoff). The F_0_ floor and ceiling range for the autocorrelation F_0_ tracker was set from 60 to 450 Hz to account for the wide range of expected F_0_s in the voice sample corpus. Similarly, the floor and ceiling search range in the CPP algorithm was also set from 60 to 450 Hz. These measures were selected for analysis of continuous speech samples based on common usage and necessity. Vocal F_0_ was chosen since it is, perhaps, the most frequently used acoustic measurement of speech samples. For measures of vocal quality, commonly used perturbation measures (such as jitter and shimmer) are not valid in continuous speech contexts. However, cepstral and spectral-based measures such as the CPP and L/H ratio have been demonstrated to be effective in characterizing vocal quality disruptions in speech contexts. They are important components of multivariate acoustic estimates of vocal severity such as the Cepstral Spectral Index of Dysphonia (18, 19) (CSID; uses both CPP as the strongest weighted factor and the L/H ratio) and the Acoustic Voice Quality Index (4) (AVQI; uses CPP as the strongest weighted factor) and, therefore, were applicable to the sentence samples being analyzed in this study. As per the manufacturer's recommendations, signal equalization was applied to all recordings prior to analyses to account for the characteristics of the HAT mouth speaker.

Statistical analyses were computed using *JASP* v. 0.19.3 (20). Due to the large number of recording methods and conditions, separate analyses were conducted for smartphones vs. tablets. For smartphones, a two-way repeated measures analyses of variance [ANOVA: five levels of recording method (GRAS Standard; iPhone + Avid-AE36; Google Pixel + Avid AE-36; iPhone Direct; Google Pixel Direct)]; and four levels of room condition (Booth; ER1; ER2; ER3) were computed to assess the presence of significant differences on the various acoustic measurements. For tablets, a two-way repeated measures ANOVA was also computed [seven levels of recording method (GRAS Standard; iPad + Avid-AE36; iPad Direct at 30 cm; iPad Direct at 45 cm; Samsung + Avid AE-36; Samsung Direct at 30 cm; Samsung Direct at 45 cm)]; and four levels of room condition (Booth; ER1; ER2; ER3). In the event of violations of sphericity, ANOVA results were evaluated using Greenhouse-Geisser corrections. For each ANOVA, effect sizes were computed using eta^2^ (*ɳ*^2^), with a small effect ≥ 0.01, a moderate effect ≥ 0.06, and a strong effect ≥ 0.14 (21). ANOVA results with negligible effects (i.e., <small effect sizes) are not discussed. *post hoc* analyses of significant ANOVAs were evaluated using Holm-Bonferroni corrections for family-wise error and *post hoc* effect sizes were evaluated using Cohen's *d* (small [0.2], medium [0.5], and large [0.8] effects) (21). Correlations between the GRAS microphone booth recordings at 2.5 cm (considered the “standard”) and measures obtained via different methods/distances/ conditions were also assessed.

## 3 Results

### 3.1 Cepstral peak processing (CPP)

**Figure 2:**
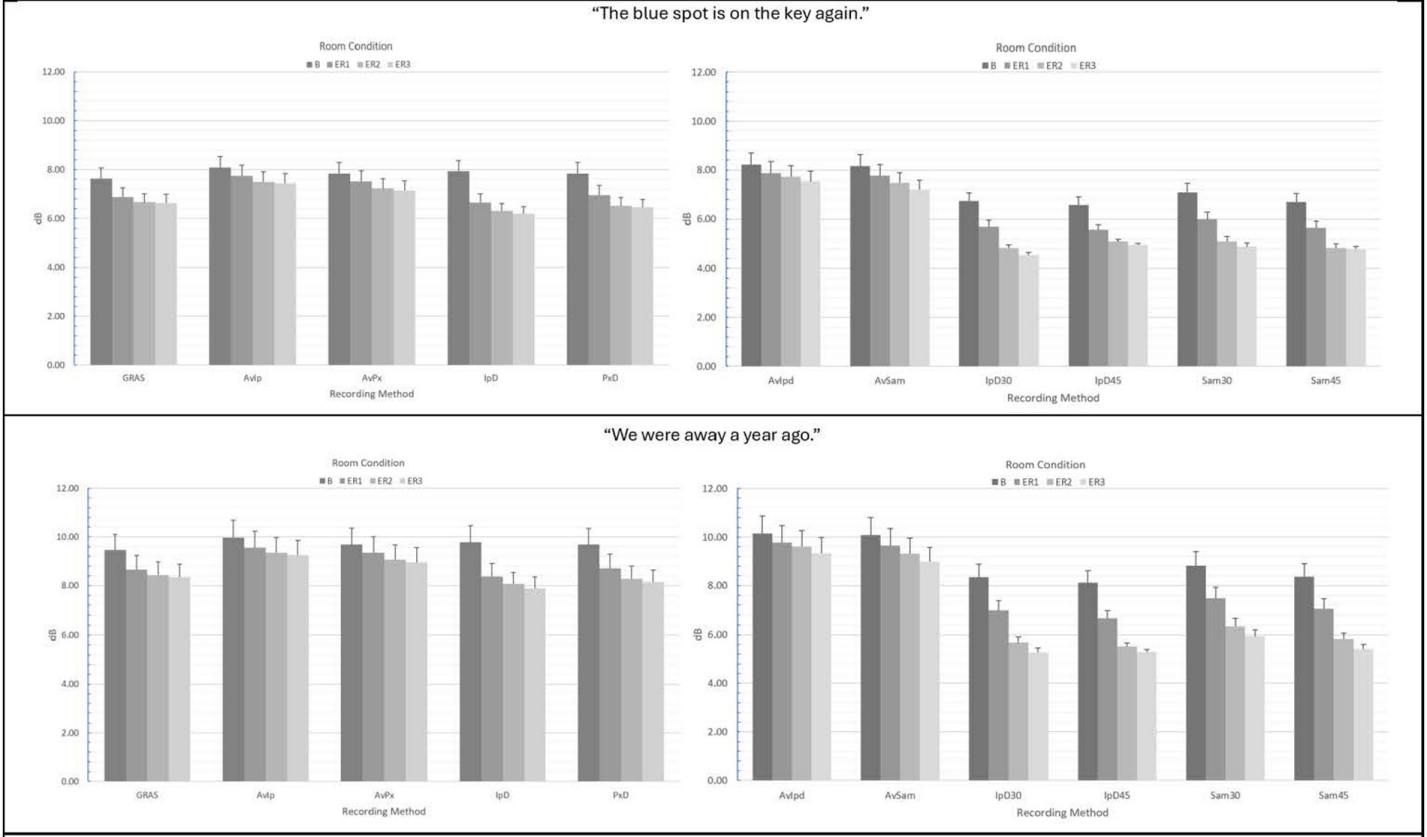
Mean CPP (dB) values and standard errors for the various recording methods and room conditions in “The blue spot is on the key again” (top) and “We were away a year ago” (bottom). GRAS, Gras 40AF Free-Field Microphone; AvIp, Avid AE-36 + iPhone 13 Pro at 2.5 cm; AvPx, Avid AE-36 + Google Pixel 6 at 2.5 cm; IpD, iPhone 13 direct at 2.5 cm; PxD, Google Pixel 6 direct at 2.5 cm; AvIpd1, Avid AE-36 +iPad 9.0 at 2.5 cm; AvSam1, Avid AE-36 + Samsung Galaxy Tab A8 at 2.5 cm; IpD30, iPad 9.0 direct at 30 cm; IpD45, iPad 9.0 direct at 45 cm; Sam30, Samsung Galaxy Tab A8 direct at 30 cm; Sam45, Samsung Galaxy Tab A8 direct at 45 cm.

For “The blue spot…” sentence, separate ANOVAs were conducted for smartphones vs. tablets. For smartphones, ANOVA main and interaction effect results for each acoustic measure are presented in Table 1 and mean CPP values and standard errors are provided in Figure 2. For the measurement of CPP, significant main effects of recording method (*p* < 0.001) and room condition (*p* < 0.001) were observed with very strong effect sizes. In addition, a significant and moderate effect size recording method × room condition interaction revealed that all of the tested recording methods produced significantly higher mean CPP (*p*'s range from 0.038 to <.001) than the GRAS standard in the booth condition, though effect sizes for these comparisons tended to be small (Cohen's d = 0.103 to 0.23). Similar findings were observed in the three noise conditions (ER1, ER2, and ER3) with the exception that the iPhone direct produced significantly lower CPP values than the GRAS standard in the ER1 condition (*p* = 0.001), and both iPhone and Pixel direct produced lower CPP values than the GRAS standard in the ER2 and ER3 conditions (*p*'s < 0.001). The effect of background noise was consistent for each recording method, with the highest CPP values observed in the booth condition, followed by successively lower CPP values in each of the ER1, ER2, and ER3 noise conditions. Regardless of any observed mean differences, highly significant Pearson's r correlations > 0.90 (*p*'s < 0.001) between the GRAS standard and the smartphones with or without microphones were observed in all conditions for all recording methods (see Table 2).

**Table 1:** ANOVA main and interaction effect results and eta^2^ (ɳ^2^) effect sizes for the various acoustic measures for the sentence “The blue spot is on the key again” obtained via smartphones with and without headset microphones.

Acoustic measure	Device	Recording method	Room condition	Recording method × room condition
CPP	Smartphones	*p* < 0.001; *ɳ*^2^ = 0.282****	*p* < 0.001; *ɳ*^2^ = 0.444****	*p* < 0.001; *ɳ*^2^ = 0.070***
	Tablets	*p* < 0.001; *ɳ*^2^ = 0.532****	*p* < 0.001; *ɳ*^2^ = 0.191****	*p* < 0.001; *ɳ*^2^ = 0.033**
CPPF_0_	Smartphones	N.S; *ɳ*^2^ = 0.015*	N.S.; *ɳ*^2^ < 0.01	N.S.; *ɳ*^2^ = 0.025*
	Tablets	*p* < 0.001; *ɳ*^2^ = 0.076***	N.S.; *ɳ*^2^ < 0.01	*p* = 0.022; *ɳ*^2^ = 0.051**
Pitch/F_0_	Smartphones	N.S; *ɳ*^2^ = 0.080***	*p* = 0.002; *ɳ*^2^ = 0.022*	*p* = 0.001; *ɳ*^2^ = 0.020*
	Tablets	*p* < 0.001; *ɳ*^2^ = 0.273****	*p* < 0.001; *ɳ*^2^ = 0.064***	*p* < 0.001; *ɳ*^2^ = 0.082***
L/H Ratio	Smartphones	*p* < 0.001; *ɳ*^2^ = 0.966****	*p* < 0.001; *ɳ*^2^ < 0.01	*p* < 0.001; *ɳ*^2^ < 0.01
	Tablets	*p* < 0.001; *ɳ*^2^ = 0.975****	N.S.; *ɳ*^2^ < 0.01	*p* < 0.001; *ɳ*^2^ < 0.01

* Small effect ≥ .01.

** Small-to-moderate effect ≥ .03 & < .06.

*** Moderate effect ≥ .06.

**** Strong effect ≥ .14.

All ANOVAs evaluated using Greenhouse-Geisser corrections.

N.S., nonsignificant.

F_0_, fundamental frequency; SD, standard deviation; HNR, harmonics-to-noise ratio; CPP, cepstral peak prominence; L/H Ratio, ratio of low (<4 kHz) vs. high (>4 kHz) spectral energy.

**Table 2:** Mean Pearson's r correlations between the various recording methods and the standard (GRAS 40AF + preamplifier + focusrite 2i2 + PC) across room conditions for selected acoustic measures of voice (all significant at *p* < .001) in the sentence “The blue spot is on the key again”. Correlation ranges across conditions are provided in parentheses.

Recording method	CPP	CPPF_0_	Pitch/F_0_	L/H ratio
AvIp	0.987 (0.982–0.992)	0.297 (−0.045–0.524)	0.962 (0.962–0.963)	0.982 (0.981–0.982)
AvPx	0.987 (0.986–0.993)	0.152 (0.006–0.295)	0.961 (0.959–0.963)	0.976 (0.976–0.977)
IpD	0.974 (0.966–0.991)	0.437 (0.270–0.627)	0.951 (0.920–0.989)	0.973 (0.971–0.984)
PxD	0.985 (0.983–0.998)	0.459 (0.346–0.716)	0.978 (0.953–0.997)	0.988 (0.984–0.994)
AvIpd	0.987 (0.982–0.991)	0.231 (0.143–0.325)	0.982 (0.980–0.984)	0.985 (0.984–0.986)
AvSam	0.991 (0.986–0.995)	0.266 (0.004–0.458)	0.976 (0.972–0.980)	0.986 (0.985–0.987)
Ipd30	0.957 (0.940–0.976)	−0.060 (−0.335–0.138)	0.919 (0.865–0.942)	0.970 (0.968–0.972)
Ipd45	0.909 (0.806–0.970)	0.253 (0.037–0.216)	0.862 (0.735–0.934)	0.951 (0.948–0.953)
Sam30	0.971 (0.958–0.995)	0.269 (−0.083–0.566)	0.941 (0.917–0.958)	0.950 (0.949–0.952)
Sam45	0.961 (0.934–0.990)	0.208 (0.037–0.481)	0.932 (0.881–0.972)	0.946 (0.945–0.948)
Across All Methods & Conditions	0.971 (0.806–0.998)	0.251 (−0.083–0.716)	0.946 (0.865–0.989)	0.979 (0.945–0.987)

AvIp, Avid AE-36 + iPhone 13 Pro at 2.5 cm; AvPx, Avid AE-36 + Google Pixel 6 at 2.5 cm; IpD, iPhone 13 direct at 2.5 cm; PxD, Google Pixel 6 direct at 2.5 cm; AvIpd1, Avid AE-36 +iPad 9.0 at 2.5 cm; AvSam1, Avid AE-36 + Samsung Galaxy Tab A8 at 2.5 cm; Ipd30, iPad 9.0 direct at 30 cm; Ipd45, iPad 9.0 direct at 45 cm; Sam30, Samsung Galaxy Tab A8 direct at 30 cm; Sam45, Samsung Galaxy Tab A8 direct at 45 cm.

CPP F_0_, Fundamental frequency (F_0_) computed from the quefrency (ms) location of the cepstral peak; Pitch/F_0_, F_0_ computed via autocorrelation in the “Analyze Periodicity | To Pitch (raw autocorrelation) … ” method of *Praat*; CPP, cepstral peak prominence; L/H Ratio, ratio of low (<4 kHz) vs. high (>4 kHz) spectral energy.

For tablets, significant main effects with very strong effect sizes for recording method (*p* < 0.001) and room condition (*p* < 0.001) were also observed (see Table 1). A significant and small-to-moderate effect size recording method × room condition interaction was also observed and indicated that tablets + the Avid headset microphone resulted in significantly higher mean CPP values vs. the GRAS standard, while recordings with tablets direct using their internal microphones resulted in significantly lower mean CPP values than the standard. Mean differences between the GRAS standard and the iPad (both 30 and 45 cm distances) and the Samsung at 45 cm were observed to have moderate effect sizes (d's = 0.560 to 0.654), whereas other mean differences were classified as small effects (d's = −0.330 to −0.368). Similar findings were observed in all three noise conditions (ER1, ER2, and ER3), with the highest CPP values for each method observed in the booth condition, followed by successively lower CPP values in each of the ER1, ER2, and ER3 noise conditions. As observed with smartphones, regardless of recording method or condition, highly significant Pearson's r correlations > 0.90 (*p*'s < 0.001) between the GRAS standard and the tablets with or without microphones (see Table 2).

Similar ANOVA results for both smartphones and tablets were observed for the “We were away a year ago” sentence (see Table 3), with the exception that overall mean CPP values for all recording methods were observed to be higher in the all-voiced context vs. the “…blue spot…” sentence containing voiced/unvoiced productions with a mixture of plosive and fricative productions. The various smartphones with and without headset microphones were again observed to produce significantly higher CPP values than the GRAS standard in the booth condition, and significantly lower mean CPP values were observed with the iPad and Samsung tablets direct. Again, regardless of any observed mean differences in CPP between methods, all correlations between the GRAS standard and the various smartphone and tablet recording methods with or without microphones were highly significant and very strong (*r*'s ≥ 0.90; *p*'s < 0.001; see Table 4).

**Table 3:** ANOVA main and interaction effect results and eta^2^ (ɳ^2^) effect sizes for the various acoustic measures for the sentence “We were away a year ago” obtained via smartphones with and without headset microphones.

Acoustic measure	Device	Recording method	Room condition	Recording method × room condition
CPP	Smartphones	*p* < 0.001; *ɳ*^2^ = 0.263****	*p* < 0.001; *ɳ*^2^ = 0.397****	*p* < 0.001; *ɳ*^2^ = 0.064***
	Tablets	*p* < 0.001; *ɳ*^2^ = 0.510****	*p* < 0.001; *ɳ*^2^ = 0.188****	*p* < 0.001; *ɳ*^2^ = 0.044**
CPPF_0_	Smartphones	N.S; *ɳ*^2^ < 0.01	N.S.; *ɳ*^2^ < 0.01	N.S.; *ɳ*^2^ = 0.023*
	Tablets	*p* < 0.001; *ɳ*^2^ = 0.186****	*p* = 0.035; *ɳ*^2^ = 0.013*	*p* = < 0.001; *ɳ*^2^ = 0.100***
Pitch/F_0_	Smartphones	N.S; *ɳ*^2^ = 0.099***	*p* < 0.001; *ɳ*^2^ = 0.026*	*p* = 0.032; *ɳ*^2^ = 0.023*
	Tablets	*p* < 0.001; *ɳ*^2^ = 0.175****	*p* < 0.001; *ɳ*^2^ = 0.093***	*p* < 0.001; *ɳ*^2^ = 0.117***
L/H Ratio	Smartphones	*p* < 0.001; *ɳ*^2^ = 0.936****	*p* = 0.007; *ɳ*^2^ < 0.01	*p* < 0.001; *ɳ*^2^ < 0.01
	Tablets	*p* < 0.001; *ɳ*^2^ = 0.906****	*p* < 0.001; *ɳ*^2^ < 0.01	*p* < 0.001; *ɳ*^2^ < 0.01

* Small effect ≥ .01.

** Small-to-moderate effect ≥ .03 & < .06.

*** Moderate effect ≥ .06.

**** Strong effect ≥ .14.

All ANOVAs evaluated using Greenhouse-Geisser corrections.

N.S., nonsignificant.

F_0_, fundamental frequency; SD, standard deviation; HNR, harmonics-to-noise ratio; CPP, cepstral peak prominence; L/H Ratio, ratio of low (<4 kHz) vs. high (>4 kHz) spectral energy.

**Table 4:** Mean Pearson's r correlations between the various recording methods and the standard (GRAS 40AF + preamplifier + focusrite 2i2 + PC) across room conditions for selected acoustic measures of voice (all significant at *p* < .001) in the sentence “We were away a year ago”. Correlation ranges across conditions are provided in parentheses.

Recording method	CPP	CPPF_0_	Pitch/F_0_	L/H ratio
AvIp	0.993 (0.987–0.996)	0.834 (0.827–0.841)	0.983 (0.978–0.985)	0.958 (0.957–0.959)
AvPx	0.994 (0.990–0.996)	0.860 (0.784–0.936)	0.974 (0.974–0.976)	0.947 (0.947–0.948)
IpD	0.984 (0.976–0.995)	0.774 (0.548–0.932)	0.990 (0.985–0.998)	0.981 (0.974–0.990)
PxD	0.992 (0.987–0.999)	0.831 (0.715–0.924)	0.994 (0.991–0.999)	0.976 (0.970–0.990)
AvIpd	0.992 (0.988–0.995)	0.851 (0.744–0.910)	0.963 (0.956–0.975)	0.974 (0.972–0.977)
AvSam	0.994 (0.989–0.997)	0.861 (0.757–0.928)	0.979 (0.972–0.990)	0.980 (0.978–0.984)
Ipd30	0.966 (0.935–0.987)	0.619 (0.571–0.689)	0.925 (0.861–0.963)	0.973 (0.947–0.987)
Ipd45	0.940 (0.870–0.987)	0.334 (0.058–0.630)	0.937 (0.886–0.962)	0.946 (0.880–0.990)
Sam30	0.985 (0.968–0.998)	0.716 (0.612–0.912)	0.963 (0.951–0.972)	0.895 (0.890–0.897)
Sam45	0.971 (0.938–0.993)	0.693 (0.526–0.789)	0.966 (0.951–0.977)	0.883 (0.879–0.886)
Across All Methods & Conditions	0.981 (0.870–0.999)	0.737 (0.058–0.936)	0.968 (0.861–0.999)	0.951v (0.879–0.990)

AvIp, Avid AE-36 + iPhone 13 Pro at 2.5 cm; AvPx, Avid AE-36 + Google Pixel 6 at 2.5 cm; IpD, iPhone 13 direct at 2.5 cm; PxD, Google Pixel 6 direct at 2.5 cm; AvIpd1, Avid AE-36 +iPad 9.0 at 2.5 cm; AvSam1, Avid AE-36 + Samsung Galaxy Tab A8 at 2.5 cm; Ipd30, iPad 9.0 direct at 30 cm; Ipd45, iPad 9.0 direct at 45 cm; Sam30, Samsung Galaxy Tab A8 direct at 30 cm; Sam45, Samsung Galaxy Tab A8 direct at 45 cm.

CPP F_0_, Fundamental frequency (F_0_) computed from the quefrency (ms) location of the cepstral peak; Pitch/F_0_, F_0_ computed via autocorrelation in the “Analyze Periodicity | To Pitch (raw autocorrelation) … ” method of *Praat*; CPP, cepstral peak prominence; L/H Ratio, ratio of low (<4 kHz) vs. high (>4 kHz) spectral energy.

### 3.2 Measures of F_0_ (method 1: CPP F_0_)

**Figure 3:**
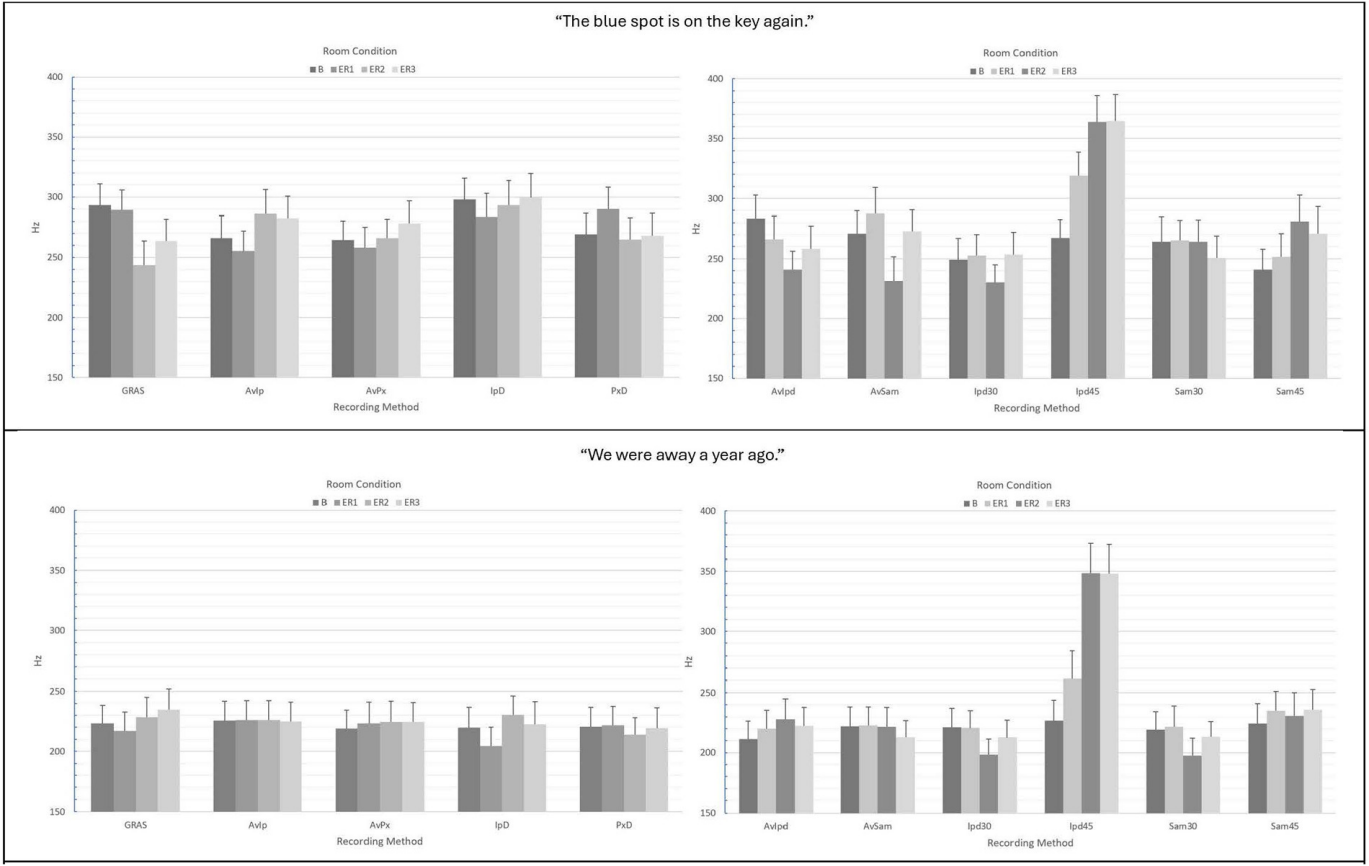
Mean CPP F_0_ (Hz) values and standard errors for the various recording methods in “The blue spot is on the key again” (top) and “We were away a year ago” (bottom). GRAS, GRAS 40AF Free-Field Microphone; AvIp, Avid AE-36 + iPhone 13 Pro at 2.5 cm; AvPx, Avid AE-36 + Google Pixel 6 at 2.5 cm; IpD, iPhone 13 direct at 2.5 cm; PxD, Google Pixel 6 direct at 2.5 cm; AvIpd1, Avid AE-36 +iPad 9.0 at 2.5 cm; AvSam1, Avid AE-36 + Samsung Galaxy Tab A8 at 2.5 cm; IpD30, iPad 9.0 direct at 30 cm; IpD45, iPad 9.0 direct at 45 cm; Sam30, Samsung Galaxy Tab A8 direct at 30 cm; Sam45, Samsung Galaxy Tab A8 direct at 45 cm.

For the measurement of CPP F_0_ in the “…blue spot…” sentence, no significant main or interaction effects were observed (see Table 1). However, Figure 3 shows a great deal of variability in mean CPP F_0_ estimates depending upon recording method and room condition. In contrast to the results for CPP, Pearson's r correlations between the GRAS standard and the smartphones with or without microphones were observed to be weak-to-moderate across conditions and recording methods (see Table 2). Similar nonsignificant ANOVA results were observed for the “We were away…” sentence (see Table 3). However, in the all-voiced context, correlations between the GRAS standard and smartphones with or without microphones were all highly significant (*p*'s ≤ 0.006) and much stronger than in the “We were away…” vs. the “…blue spot …” context (see Table 4). The weakest observed correlation (*r* = 0.548) with the GRAS standard was observed for the iPhone direct in the ER1 condition. Review of data indicated that this weaker correlation was due to four outliers representative of increased dysphonic voice.

For CPP F_0_ analysis via tablet recordings of the “…blue spot…” sentence, a significant main effect of recording method (*p* < 0.001), as well as a significant interaction of recording method x room condition (*p* = 0.022) were observed (see Table 1). Figure 3 shows a great deal of variability in mean CPP F_0_ estimates depending upon recording method and room condition, and substantially higher estimates of CPP F_0_ for the iPad direct at 45 cm in noise conditions (ER1, ER2, and ER3). Similar to the smartphone analyses of CPP F_0_ in the “…blue spot…” context, Pearson's r correlations between the GRAS standard and the tablets with or without microphones were again observed to be weak-to-moderate across conditions and recording methods (see Table 2). Similar ANOVA results were observed for the “We were away…” sentence (see Table 3). However, in the all-voiced context, correlations between the GRAS standard and tablets with or without microphones were much stronger than in the “…blue spot …” context (see Table 4). The weakest observed correlation (*r* = 0.058) was observed for the iPad direct at 45 cm in the ER2 condition. Review of data indicates that the extremely weak correlation was due to a large number of subjects (15/24; 62.5%) who were assigned a CPP F_0_ value that approximated the upper limit of the CPP search limit of 450 Hz.

### 3.3 Measures of F_0_ (method 2: autocorrelation F_0_ tracker)

**Figure 4:**
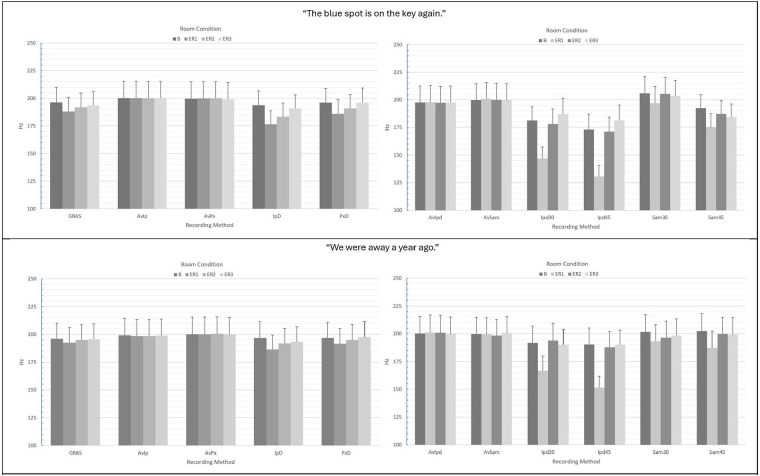
Mean Pitch/F_0_ (Hz) values and standard errors computed via autocorrelation for the various recording methods in “The blue spot is on the key again” (top) and “We were away a year ago” (bottom). GRAS, GRAS 40AF Free-Field Microphone; AvIp, Avid AE-36 + iPhone 13 Pro at 2.5 cm; AvPx, Avid AE-36 + Google Pixel 6 at 2.5 cm; IpD, iPhone 13 direct at 2.5 cm; PxD; Google Pixel 6 direct at 2.5 cm; AvIpd1, Avid AE-36 +iPad 9.0 at 2.5 cm; AvSam1, Avid AE-36 + Samsung Galaxy Tab A8 at 2.5 cm; IpD30, iPad 9.0 direct at 30 cm; IpD45, iPad 9.0 direct at 45 cm; Sam30, Samsung Galaxy Tab A8 direct at 30 cm; Sam45, Samsung Galaxy Tab A8 direct at 45 cm.

For smartphones, ANOVA main and interaction effect results using the pitch/F_0_ autocorrelation method are presented in Tables 1, 3 and mean pitch/F_0_ values and standard errors are provided in Figure 4. For the measurement of pitch/F_0_ in the “…blue spot…” sentence, no significant main effect of recording method was observed, though the effect size was moderate (*p* = 0.131; *ɳ*^2^ = 0.080; see Table 1). A significant main effect of room condition was observed (*p* < 0.001), as well as a significant interaction between recording method x room condition (*p* < 0.001) with a small effect size. Figure 4 shows greater stability in mean F_0_ estimates vs. those observed for CPP F_0_. Following Bonferroni-Holm corrections, there was no significant difference in autocorrelation-based F_0_ estimation between the GRAS standard and any of the smartphones with or without headset microphones in any of the conditions. Within recording methods, the differences in mean pitch/F_0_s observed for the iPhone direct in ER1 vs. ER3 was marginally significant (*p* = 0.063). In contrast to the results for CPP F_0_, Pearson's r correlations between the GRAS standard and the smartphones with or without microphones for pitch/F_0_ were observed to be very strong across conditions and recording methods (see Table 2). Similar ANOVA results were observed for the “We were away…” sentence, with no significant difference between the GRAS standard and any of the smartphones with or without headset microphones and no significant differences observed within recording methods (see Table 3). Correlations between the GRAS standard and smartphones with or without microphones in the all-voiced context were all highly significant (*p*'s ≤ 0.001) and consistently very strong across conditions and recording methods (see Table 4).

For autocorrelation F_0_ analysis via tablet recordings of the “…blue spot…” sentence, significant main effects of recording method and room condition, as well as a significant interaction of recording method × room condition were observed (all at *p* < 0.001; see Table 1). Analysis of the significant interaction effect showed that iPad direct recordings at both 30 and 45 cm resulted in significantly lower estimates of pitch/F_0_ vs. the GRAS standard in the ER1 condition only (*p*s < 0.001). iPad direct methods also showed significant differences in pitch/F_0_ estimates between the booth and ER1 conditions. Figure 4 shows the increased variability in tablet direct estimates of pitch/F_0_. Similar to the smartphone analyses of pitch/F_0_, Pearson's r correlations between the GRAS standard and the tablets with or without microphones in the “…blue spot…” context were observed to be generally very strong across conditions and recording methods (see Table 2), with the lowest observed correlation observed for the iPad direct at 45 cm in the ER1 condition. Similar ANOVA results were observed for the “We were away…” sentence (see Table 3). However, in the all-voiced context, analysis of the significant interaction effect showed a significant difference in pitch/F_0_ only between iPad direct recordings at 45 cm vs. the GRAS standard in the ER1 condition. The iPad direct at 30 cm showed a marginally significant difference (*p* = 0. 069) between booth and ER1 pitch/F_0_ estimates, while the same comparison for iPad direct at 45 cm was highly significant (*p* = 0.001). Correlations between the GRAS standard and tablets with or without microphones in the “We were away…” context were also very strong (see Table 4), with the weakest observed correlation observed for the iPad direct at 30 cm in the ER1 condition.

### 3.4 Low/high (L/H) ratio

**Figure 5:**
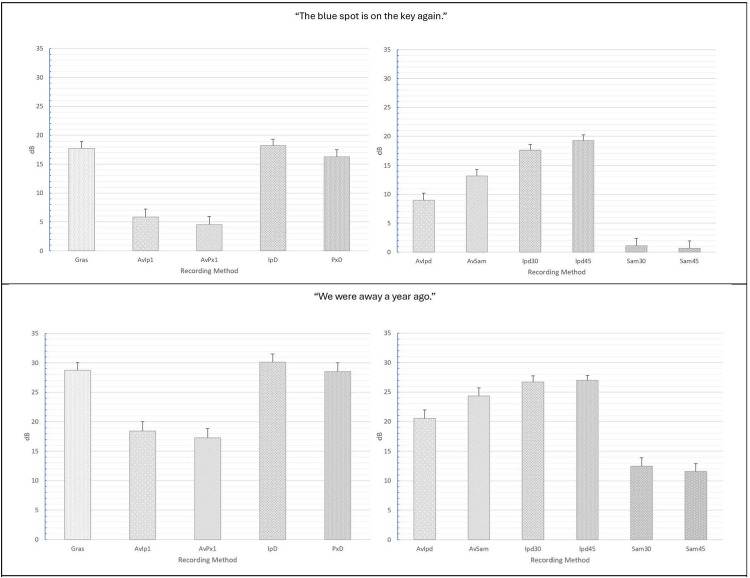
Mean L/H ratio (dB) values and standard errors computed for the various recording methods in “The blue spot is on the key again” (top) and “We were away a year ago” (bottom). GRAS, GRAS 40AF Free-Field Microphone; AvIp, Avid AE-36 + iPhone 13 Pro at 2.5 cm; AvPx, Avid AE-36 + Google Pixel 6 at 2.5 cm; IpD, iPhone 13 direct at 2.5 cm; PxD, Google Pixel 6 direct at 2.5 cm; AvIpd1, Avid AE-36 +iPad 9.0 at 2.5 cm; AvSam1, Avid AE-36 + Samsung Galaxy Tab A8 at 2.5 cm; IpD30, iPad9.0 direct at 30 cm; IpD45, iPad 9.0 direct at 45 cm; Sam30, Samsung Galaxy Tab A8 direct at 30 cm; Sam45, Samsung Galaxy Tab A8 direct at 45 cm.

For the analysis of L/H ratio in the “…blue spot…” sentence, a significant strong effect of recording method was observed (*p* < 0.001; see Table 1). Though significant effects of room condition and recording method × room condition were also observed (*p* < 0.001, respectively), effect sizes were negligible (*ɳ*^2^ < 0.01). *post-hoc* analysis of the main effect of recording condition showed significantly greater mean L/H ratios for the GRAS standard vs. Avid + iPhone, Avid + Pixel, and Pixel direct (*p* < 0.001 for all comparisons). Regardless of any observed significant differences in mean L/H ratio, Pearson's r correlations between the GRAS standard and the smartphones with or without microphones were observed to be very strong across conditions and recording methods (see Table 2). Similar ANOVA results were observed for the “We were away…” sentence (see Table 3), with *post-hoc* analysis again showing significantly lower L/H ratio estimates for the smartphones + Avid headset vs. the GRAS standard. In addition, the mean L/H ratio estimate from the iPhone direct was observed to be significantly higher than the GRAS standard and there was no observed significant difference between the Pixel direct vs. the GRAS standard (see Figure 5). Again, consistently strong Pearson's r correlations between the GRAS standard and the smartphones with or without microphones were observed across conditions and recording methods (see Table 4).

Similar findings were observed for L/H ratio analyses using tablets with and without headset microphones. In the “…blue spot…” sentence, the main effect of recording method was again highly significant (*p* < 0.001) with a very strong effect size (see Table 1). *post-hoc* analysis of the main effect of recording condition showed significantly lower mean L/H ratios for tablets with the Avid headset microphone as well as for the iPad direct at 45 cm and the Samsung tablet direct at both 30 and 45 cm. Similar results were observed for L/H ratio analyses using tablets with and without headset microphones in the “We were away…” context, with all recording methods (all tablets with and without the Avid microphone at all tested distances) resulting in significantly reduced L/H ratios vs. the standard (see Figure 5). Regardless of observed differences in mean L/H ratios, all recording methods correlated extremely strongly with the GRAS standard in both the “…blue spot…” and the “We were away…” contexts (see Tables 2, 4). Slightly lower correlations were observed between the standard and Samsung direct measures of L/H ratio at both 30 and 45 cm and were due to a single highly dysphonic voice sample that was computed as having a particularly low L/H ratio.

## 4 Discussion

The Bridge2AI-Voice Consortium is focused on the development of affordable and accessible voice data to support voice AI research. The goal is to provide a database of quality recordings representative of typical, nondysphonic voice, as well as voices demonstrating a variety of vocal, respiratory, neurological, and mood disorders. An additional goal is to collect a database of voices of children with common childhood disorders. With these goals in mind, the data acquisition team performed a series of experiments designed to establish recording procedures to be used with a wide variety of typical and dysphonic voices in research labs and clinical settings, as well as in quiet environments outside of the clinic. This process entailed evaluating the impact of recording devices, including low-cost microphones, smartphones, and tablets on the measurement of various acoustic parameters slated for use in the development of potential vocal biomarkers of disease. Our initial experiments have focused on isolated vowel productions, which provide a vocal signal that is representative of the biomechanics of the larynx within a static vocal tract. The current experiment considers the impact of sentence productions on the measurement of several acoustic parameters. Sentence-level stimuli introduce the impact of both speech signal complexity, articulator movements (i.e., tongue, jaw, lips, etc.), and rapid variations in glottal configuration (voiced vs. unvoiced productions) and laryngeal adjustments for pitch and loudness on the acoustic signal. As such, how one produces a sentence may reflect the impact of a vocal disorder on the production of speech (1–3). Measures of voice quality acceptable for vowels (i.e., perturbation measures such as jitter, shimmer, HNR) are not valid in sentence-level recordings (1–5). For this reason, we assessed the influence of recording device and noise on a set of previously validated sentence-level measures related to vocal pitch and quality: CPP, CPP F_0_, F_0_, and L/H ratio.

### 4.1 Impacts on CPP values

CPP (dB) is a well-accepted “objective measure of breathiness and overall dysphonia” in the voice (15, 22, 23). Increased CPP values are expected in the highly periodic voice productions of nondysphonic speakers. In contrast, lower CPP values are consistent with increased aperiodicity (possibly due to irregularity in vocal fold closure patterns and/or the presence of additive noise) that may result in the perception of hoarseness, harshness, or breathiness (23, 24). Unlike traditional perturbation measures such as jitter, shimmer and HNR, the CPP has been shown to provide valid measures of dysphonia in both vowel and sentence contexts and in voice samples representative of more than mild dysphonia. This is because the CPP is not dependent upon F_0_ tracking and the accurate identification of cycle boundaries (18) and therefore can provide accurate estimates of vocal quality in both sentence-level and vowel productions, as well as in highly dysphonic voice samples. In comparison to the GRAS microphone standard, statistical analyses assessing changes in mean CPP (dB) values from the two sentences revealed higher CPP values when smartphone and tablet recordings were made with the headset microphone, while recordings made directly using the built-in smartphone and tablet microphones resulted in lower CPP values. CPP measures were observed to be quite sensitive to background noise conditions, with the main effect of room condition particularly prominent and consistent across recording methods. Since the Avid AE-36 headset microphone is noise-canceling (i.e., employing two separated microphones, with one used to pick up the voice signal, the other [generally located on the opposite side of the microphone housing] used to pick up background noise, and digital algorithms used to “subtract” the background noise from the recordings) (12), it is not surprising that CPP values were higher for the recordings using the headset microphone. In contrast, the built-in MEMS microphone used in the smartphone and tablet recordings direct were more prone to background noise interference, particularly at the larger mouth to microphone distances used with tablet recordings. The strong main effects of recording method observed in the analysis of CPP clearly indicate that measured CPP values using different recording methodologies are not necessarily interchangeable. Therefore, normative data for measures, such as CPP, must be evaluated with knowledge of both the recording methodology used and the background noise conditions by which the data were collected. Clearly, both normative and dysphonic expectations for mean CPP may differ substantially from published norms if very different recording methodologies and room conditions are used. However, the strong correlations for both sentences (mean r's = 0.971 and 0.981) between the CPP values obtained with the GRAS recording method and all other recording methods indicate that all of the recording methodologies tested in this study were able to track the wide range of voice qualities and types assembled in the 24-voice typical and dysphonic voice corpus. In addition, the strong linear relationships observed between the various recording methodologies and the GRAS standard indicate that measures from one method (e.g., smartphone direct) may be transformed to that of another method (e.g., GRAS standard) using linear predictive formulae. In practice, the results of this and previous studies (8, 9) indicate that recording methodologies such as those used in this study may be effective in group comparisons (e.g., typical vs., disordered) or to track pre- vs. post-treatment outcome, *as long as the same recording methodology is used in similar recording conditions.*

Differences in CPP measurements were noted across sentences, with higher mean CPP values observed in the “We were away” vs. the “blue spot” sentence. This finding is due to differences in the phonetic context of the sentences. The first sentence “The blue spot is on the key again” contains speech sounds that are not voiced, like the /sp/ in “spot” or the /k/ in “key”. On the other hand, “We were away” contains only voiced sounds and vowels, so the abrupt voiced to or from unvoiced transitions in speech sound productions are mitigated (4). The differences in sentence construction are designed to elicit different aspects of voice quality (11). However, separate normative and dysphonic expectations will be expected for different sentences and associated phonetic contexts. *Therefore, group comparisons or within-subject treatment outcomes comparisons regarding CPP must be evaluated using the same sentence.*

### 4.2 Measurement of F_0_ (Hz)

A commonly used acoustic measure in voice evaluation is F_0_, which is a measure of the number of vocal fold cycles of vibration per second and is generally perceived as the pitch of a voice. This study employed two different methods to track F_0_ in the sentence context. The first, CPP F_0_, was the F_0_ determined from the quefrency location (in ms) of the observed cepstral peak, while the second method was the autocorrelation method recommended in *Praat* for use in tracking intonation patterns. While a number of previous studies have reported that measures of F_0_ are fairly robust to variations in recording methodology and to the effects of background noise (9, 25, 26), the results of this study indicate that this assumption is not necessarily true in sentence contexts. *The results of this study indicate that the method of F_0_ estimation can have a substantial effect on the accuracy of F_0_ measures and may be affected by both recording methodology, background noise condition, and speech context.* In particular, F_0_ tracking using the cepstrum (CPP F_0_) was detrimentally affected by recording methodology, with the iPad at a 45 cm mouth-to-microphone distance showing extreme deviations in F_0_ estimation vs. the GRAS standard, particularly in the ER1, ER2 and ER3 clinic room noise conditions. It is unclear as to why this deviation was primarily restricted to the iPad methodology. Due to available funding, multiple versions of the iPad were not able to be tested. Therefore, it may be possible that this finding was due to some potential characteristic unique to our tested model.

Though nonsignificant, all methods showed a strong effect of room condition on CPP F_0_, with variability in F_0_ estimation observed between all room conditions across methods. Perhaps more troubling is the observation that the observed correlations between the various smartphone and tablet recording methodologies vs. the GRAS standard were consistently weak in the “blue spot” sentence (mean *r* = 0.251), indicating a weak relationship between that the CPP F_0_ estimates from the GRAS standard vs. similar estimates measured via these other methods. While correlations improved considerably in the “We were away…” context (mean *r* = 0.737), these observations indicate that F_0_ estimation from the cepstrum is highly susceptible to noise, both from room condition background noise and from noise inherent within dysphonic voice samples themselves. In these cases, the selected CPP is affected by increased amplitude noise and is not necessarily reflective of underlying periodic energy concentrated in the F_0_ and harmonics. *It is possible that the influence of recording-related noise is nonlinear across varying levels of dysphonia* (18) *and, as observed in this study, the influence of recording methodology and/or room condition background noise may be more detrimental when it interacts with voice signals that are already highly degraded by severe levels of dysphonia.*

Though significant recording method × room condition interactions were observed in both sentence contexts for smartphones and tablets, the second method of F_0_ tracking using autocorrelation was observed to be much more robust to recording methodology and room condition vs. CPP F_0_. Autocorrelation is a measure of the degree of correlation of a signal between two successive time intervals (the original signal vs. a lagged version) and determines how similar sequential data points are over time. Highly periodic voice signals will show high autocorrelation peaks vs. low autocorrelation peaks in highly dysphonic and/or noisy signals. As in CPP F_0_, iPad direct recordings (at both 30 and 45 cm) resulted in significantly lower estimates of pitch/F_0_ vs. the GRAS standard in the ER1 condition as well as increased variability in F_0_ estimation between room conditions. This increased variability was evident in all tablet conditions (both iPad and Samsung) when recordings were made directly using the built-in MEMS microphones at 30 and 45 cm, as well as in smartphone direct recordings (see Figure 4). We speculate that these findings, i.e., those illustrating that recording methodology and room condition can have, in certain cases, a significant effect on F_0_ estimation, differ from previous reports (9, 23, 24) due to the type of voice sample elicited. The previous studies which had reported on the robustness of F_0_ estimation to recording methodology and background noise had all used sustained vowel samples in which there is relative consistency in pitch and loudness and a target of continuous phonation. Such contexts make it much easier for algorithms to track F_0_ vs. speech samples in which rapid variations in pitch, loudness, and voiced vs. unvoiced transitions naturally occur. Fortunately, and in contrast to CPP F_0_ measures, F_0_ estimates via autocorrelation were observed to consistently correlate very strongly with the GRAS standard, regardless of recording methodology, room condition, or sentence context (“blue spot…” mean *r* = 0.946; “We were away…” mean *r* = 0.968), indicating that autocorrelation F_0_ estimates from the smartphone and tablet methods were highly predictive of similar estimates from the GRAS standard.

For both smartphones and tablets, the strength of the correlations between both CPP F_0_ and autocorrelation F_0_ estimates from the GRAS standard vs. the smartphone and tablet conditions were stronger for the “We were away” sentence than the “blue spot” sentence. Because, in this study, CPP F_0_ was measured without any application of voicing activity detection (VAD), CPP estimates were obtained not just from voiced portions of the voice signals, but also from both unvoiced and highly dysphonic segments, and it is probably the spurious F_0_ estimates from these segments that resulted in the increased variability in CPP F_0_ estimation observed in the “blue spot” sentence. For this experiment, VAD was not applied in the cepstral analyses because VAD can inadvertently remove dysphonic segments that are actually the focus of the analysis. Therefore, the “We were away…” sentence, which is comprised of relatively continuous voicing without the intrusion of voiced to unvoiced transitions, provided CPP F_0_ estimates that were not as variable. F_0_ estimates using autocorrelation only provide F_0_ estimates for autocorrelation peaks that occur above a predetermined threshold that is used for voiced vs. unvoiced decision making. Therefore, autocorrelation F_0_ tracking will generally not provide F_0_ estimates for clearly unvoiced segments or highly dysphonic segments of a voice sample, resulting in less variability in F_0_ estimation. Users of these types of analyses should recognize the strengths and limitations of these various methods. CPP is a highly effective measure of noise in the voice signal (both inherent in the voice signal and from external sources) which results in a strong measure of dysphonia but will result in increased variability in F_0_ estimation with increased noise levels. On the other hand, autocorrelation will produce less variability in F_0_ estimation in both voiced and unvoiced contexts, but at the expense of potentially removing data segments that may be reflective of the dysphonia that we actually want to measure. *It is notable that the use of a headset microphone tends to reduce variability in F_0_ estimates in both CPP F_0_ and autocorrelation F_0_ estimation since the detrimental effects of room condition background noise are reduced vs. use of the smartphone or tablet microphones direct.*

### 4.3 Usefulness of low/high ratios

The last measure that was tested was L/H ratio [low vs. high spectral energy using a 4 kHz cutoff (27)]. It is frequently used along with cepstral measures (like CPP) as a measure of spectral tilt. Researchers have shown that individuals that present with breathy voices or increased vocal tension often demonstrate a lower L/H ratio (24, 27) due to the presence of increased high frequency energy from additive noise (e.g., from air escape between the vocal folds) and/or enhanced high frequency energy due to pressed phonation.

Results indicated that the L/H ratio was affected by recording condition, but not room condition background noise. These findings suggest that it is a good complement to the information provided by CPP. In the “blue spot” sentence, L/H ratio results were significantly influenced by recording method, with recordings made with the Avid headset microphone and the smartphones resulting in measures of L/H ratio that were significantly lower than the GRAS standard and the smartphone direct recordings. Similar findings were noted with the measurements from the “We were away” sentence, with the exception of the iPhone direct recordings where the L/H ratios were higher. The frequency response of the microphones in these devices seems to be a contributory factor to the variability in these measurements. *Microphones such as the Avid AE-36 and the built-in microphone used in the Samsung tablet which have a high frequency emphasis will tend to produce recordings that have lower L/H ratios vs. those that have either a flatter response or have balanced regions of emphasis (i.e., regions of frequency emphasis both below and above the 4 kHz cutoff used in the L./H ratio calculation)* (see Figure 5)*.* Differences across various recording methods could easily be adjusted with corrective equalization by adjusting the spectral characteristics of the recording with the microphone being used (e.g., smartphone or tablet with or without headset microphone) to better match a standard (e.g., the flat frequency response GRAS 40AF Free-Field Microphone used as the standard in this study). The ability to correct these measurements to the standard is further supported by their strong correlations with the GRAS standard (mean *r*'s > 0.90; see Tables 2, 4), with all methods correlating well with the GRAS standard.

## 5 Limitations and conclusions

There are several limitations to this study. First, the cepstral peak and F_0_ tracking floor and ceiling parameters were fixed at 60 to 450 Hz for all samples and recording methods used in this study. This allowed for the isolation of the effects of recording method and background noise. However, manipulation of analysis parameters for different voice samples and different conditions may have resulted in improved analysis results (e.g., for F_0_ tracking) for certain recording methods. Second, while the consistency of frequency response for multiple Avid AE-36 microphones has been reported (8, 9), we were unable to test multiple versions of the smartphones and tablets used in this study to note consistency of frequency response and recording quality. It is possible that the highly variable F_0_ tracking results for the iPad direct at 45 cm (see Figures 3, 4), as well as the particularly low L/H ratio results for the Samsung Galaxy Tab 8 (see Figure 5) may not be characteristic of other similar model tablets. Though potentially expensive, future studies that examine acoustic estimates of voice obtained from smartphone and/or tablet recordings should evaluate consistency of results among multiple examples of the same device.

The current findings illustrate that different recording methods can produce significantly different acoustic analysis results for the voice quality measures used in sentence analysis. Microphone characteristics (e.g., frequency response; use of noise cancellation), mouth-to-microphone distances, and background noise conditions all can have significant effects on acoustic results using sentence-level materials. However, in the cases of CPP, Pitch/F_0_ estimation via autocorrelation, and L/H ratio, different recording methods were observed to be highly correlated with the GRAS standard (in most cases, r's substantially greater than 0.90). As such, all recording methods (smartphones and tablets, with and without headset microphones) were able to track the acoustic expectations observed in the highly diverse typical to highly dysphonic voice samples used in this study. The greatest variability in acoustic measurement results was observed in the use of tablets direct (i.e., using their built-in MEMS microphones) at increased mouth-to-microphone distances. While convenient, recording directly into a tablet at increased mouth-to-microphone distances of 30 to 45 cm allows background noise to substantially affect recording quality and acoustic estimates of voice, with decreased measures of CPP and highly variable measures of CPP F_0_ observed vs. the booth condition. In contrast, recordings using close mouth-to-microphone distances (e.g., smartphones direct or preferably with a headset microphone at a short mouth-to-microphone distance, such as the 2.5 cm in this study) reduce the detrimental effects of background noise and potential reverberation, resulting in higher CPP estimates and a tendency for less variability in F_0_ tracking. Therefore, when recording conditions or available funding does not allow for voice recordings to be collected via instrumentation that meet established guidelines [e.g., Patel et al. (15)], mobile devices such as smartphones and tablets, ideally with attached headset microphones, may be used to provide acoustic measures for documenting the presence of dysphonia.

Researchers should always describe their data collection protocols when comparing datasets, as well as when releasing an audio dataset, to allow accurate interpretation of data. It is important to recognize that this project focused on the assessment of spectral and cepstral analyses of sentence-level materials. There are other vocal or acoustic biomarkers that go beyond these traditional acoustic features. For example, linguistic or paralinguistic biomarkers may not be as sensitive to recording conditions. Further work is needed to compare accuracy of other speech, linguistic and paralinguistic biomarkers in different recording conditions.

## Data Availability

The raw data supporting the conclusions of this article will be made available by the authors, without undue reservation.
